# How safe are new drugs? Market withdrawal of drugs approved in Canada between 1990 and 2009

**Published:** 2014-01-28

**Authors:** Joel Lexchin

**Affiliations:** Joel Lexchin, MSc, MD, is a Professor in the School of Health Policy and Management at York University, a staff physician in the Emergency Department of the University Health Network, and an Associate Professor in the Department of Family and Community Medicine, University of Toronto, Toronto, Ontario.

## Abstract

**Background:**

Studying drugs withdrawn from the market for safety reasons can help in evaluating the strengths and weaknesses of the pre- and post-market safety evaluation systems. This study considered 2 questions: Has there been a change over time in the percentage of new drugs that are eventually withdrawn because of safety reasons? How long are new drugs on the market before their serious safety problems are recognized?

**Methods:**

All drugs approved between 1 January 1990 and 31 December 2009 and subsequently withdrawn for safety reasons (until 1 October 2013) were identified, and the generic name, date of approval, and date of withdrawal were recorded. The total number of drugs approved over the same period was obtained from annual Health Canada reports. The percentages of new active substances approved in the 5-year periods 1990–1994, 1995–1999, 2000–2004, and 2005–2009 and eventually withdrawn were compared using the χ^2^ test. The time between approval and withdrawal was calculated in days.

**Results:**

Of the 528 new drugs approved over the period of interest, a total of 22 (4.2%) were eventually withdrawn. Between 3.9% and 4.4% of the drugs approved in each 5-year period were eventually withdrawn (χ^2^ = 0.04, p = 0.99 for difference among 5-year periods). The median time between approval and withdrawal was 1271 days (interquartile range 706–2876).

**Interpretation:**

One explanation for the finding of no difference in the percentage of drugs approved in the four 5-year periods that were eventually withdrawn is the lack of any change in the rigour of the premarket evaluation system and the postmarket surveillance systems. The 1271-day median time between Notice of Compliance and withdrawal emphasizes the need to be particularly cautious in prescribing new drugs early in their life cycle.

When a new active substance (a molecule that has never been marketed in Canada in any form) receives its Notice of Compliance (i.e., marketing approval), relatively little is known about its safety profile. This situation exists for a number of reasons, including the relatively homogeneous nature of the population enrolled in premarket clinical trials, the use of pre-randomization run-in periods, the short-term nature of many trials, and the relatively small number of patients in these trials.^1^,^2^

One measure of the limited initial information about safety is the number of new active substances—just under 1 in 4—for which Health Canada eventually issues a serious safety warning (a warning in bold black lettering and/or a boxed warning) or that must be withdrawn from the market because of safety concerns.^3^ Drugs in the latter category are the ones with the most serious safety issues, because whatever their therapeutic benefits, they are too dangerous to remain on the market. Looking at this group of drugs can provide insights into the strengths and weaknesses of both the pre- and the post-market safety evaluation systems.

Since the early 1990s, there have been a number of changes to the Canadian regulatory system that may have affected its ability to detect safety problems before drugs are approved and to monitor the safety of drugs once they are on the market. In 1994, Health Canada instituted a system of cost recovery from pharmaceutical companies to cover part of the operating costs of the drug regulatory system. Critics have charged that user fees have redirected the orientation of Health Canada away from drug safety toward faster processing of new drug applications and a higher approval rate.^4^ New drug approval times in Canada declined significantly after 2006,^5^ and shorter times for approving new drug applications have been linked to more postmarket safety problems.^3^,^6^ In 2002, Health Canada reorganized its postmarket safety system, creating the Marketed Health Products Directorate, and dedicated $7 million in new funding in fiscal year 2002/2003 to strengthen post-market surveillance activities concerned with safety and effectiveness.^7^ Between 2004 and 2010, the ratio of funding and personnel allocated by Health Canada to the directorates that review new drug applications to the funding and personnel for the Marketed Health Products Directorate improved from about 7:1 to 3.5:1.^8^

Specifically, this study considered 2 questions: Has there been a change in the percentage of new active substances approved in 5-year periods between the start of 1990 and the end of 2009 that are eventually withdrawn because of safety reasons? How long are new active substances on the market before their serious safety problems requiring withdrawal are recognized? In addition, 2 secondary questions were examined: For drugs that are eventually withdrawn that first receive a serious safety warning, what is the period between approval of the drug and its first serious safety warning, and what is the period between the safety warning and eventual withdrawal?

## Methods

### Identification and classification of drugs withdrawn from the Canadian market

A list of all drugs withdrawn from the Canadian market between 1 January 1990 and 1 October 2013 and the reason for withdrawal was compiled from Lexchin^9^ and Health Canada's MedEffect website (www.hc-sc.gc.ca/dhp-mps/medeff/advisories-avis/prof/index-eng.php). Withdrawals of specific lots of a drug due to manufacturing problems were excluded. This list was narrowed to those approved from 1 January 1990 to 31 December 2009 using the Notice of Compliance website (www.hc-sc.gc.ca/dhp-mps/prodpharma/notices-avis/index-eng.php). For each of these drugs the following information was recorded: generic name, date of Notice of Compliance, and date of and reason for market withdrawal. Each drug was classified at the third level of the World Health Organization's Anatomical Therapeutic Chemical system.^10^

The total number of new active substances approved in consecutive 5-year periods (1990–1994, 1995–1999, 2000–2004, 2005–2009) was obtained from the annual reports of the Therapeutic Products Directorate and the Biologic and Genetic Therapies Directorate (available by contacting the directorates directly at publications@hc-sc.gc.ca).

### Identification of serious safety warnings before withdrawal

For each drug that was withdrawn, the MedEffect website was used to identify whether the product had received a serious safety warning before its withdrawal and, if so, the date of the warning. Serious safety advisories issued because of misuse of a drug (e.g., an unapproved use) or medication errors (e.g., neglecting to remove a transdermal patch before applying a second one) were excluded. When necessary, notices on the MedEffect web site were supplemented by searching the product name in the Drug Product Database (http://webprod5.hc-sc.gc.ca/dpd-bdpp/index-eng.jsp). For example, a notice that one product, gatifloxacin, had been withdrawn from the market never appeared on the MedEffect website, and the withdrawal was confirmed only on the Drug Product Database website.

### Discrepancies in data sources

For 4 drugs, the data sources yielded conflicting information. Troglitazone was approved but never marketed in Canada because of a dispute about its introductory price. There was no information about revocation of its Notice of Compliance on the MedEffect website. The drug was removed from the US market in March 2000, and 15 March 2000 was arbitrarily used as its withdrawal date in Canada. It was retained in the analysis because it was approved and then later shown to have side effects serious enough that it was withdrawn. The announcement about the withdrawal of cisapride made reference to a safety warning in February 2000 but the exact date was not stated; the date of the safety warning was therefore arbitrarily set to 14 February. Amphetamine salts were withdrawn on 9 February 2005 but allowed back on the market on 24 August 2005 and were therefore not included in the list of drugs withdrawn for safety reasons. Nesiritide was withdrawn by the company marketing the product but no reason was given, so it was also excluded from analysis.

### Statistical analysis

The percentages of new active substances withdrawn in the four 5-year periods were compared using the χ^2^ test (AcaStat 8.2.3, AcaStat Software). The following time periods were calculated in days: from Notice of Compliance to first serious safety warning, from Notice of Compliance to withdrawal, and from first serious safety warning to withdrawal. These calculations were done with Excel 2011 for Macintosh (Microsoft Inc.)

### Funding and ethics approval

There was no funding for this study, and no ethics approval was required because all of the information came from publicly available databases.

## Results

A total of 528 new active substances were approved between 1 January 1990 and 31 December 2009; of these, 22 (4.2%, 95% confidence interval 2.5%–5.9%) were withdrawn (Table 1). Nineteen of the drugs were withdrawn for safety reasons and 2 (drotrecogin alfa and idebenone) were withdrawn because of a negative benefit-to-harm ratio rather than a specific safety problem. The reason for withdrawal of ceftobiprole was vaguely given as concerns regarding the conduct of clinical trials, and it is not clear that safety was an issue.

**Table 1:** Drugs eventually withdrawn for safety reasons relative to all new active substances approved in various 5-year periods, from 1 January 1990 to 31 December 2009*

Variable	Approval period; no. or % of products				
	1990–1994	1995–1999	2000–2004	2005–2009	Overall (1990–2009)
No. of new active substances approved	129	166	119	114	528
No. of products eventually withdrawn†	5	7	5	5	22
% of new active substances withdrawn (95% CI)	3.9 (0.6–7.2)	4.2 (1.2–7.3)	4.2 (0.6–7.8)	4.4 (0.6–8.2)	4.2 (2.5–5.9)

CI = confidence interval.

* No difference among 5-year periods (χ^2^ = 0.04, *p* = 0.99).

† Withdrawals anytime between 1 January 1990 and 1 October 2013.

Between 3.9% and 4.4% of the drugs approved in each 5-year period were eventually withdrawn (χ^2^ = 0.04, p = 0.99 for differences among periods) (Table 1).

Of the 22 drugs withdrawn, 11 first had a serious safety warning and 11 did not (Table 2). The median time between the Notice of Compliance and withdrawal was 1271 days (interquartile range 706–2876). For the 11 drugs with a prior safety warning, the median time between Notice of Compliance and the warning was 907 days (interquartile range 196–1525), and the median time between the warning and withdrawal was 329 days (interquartile range 119–893). Two of the drugs (sitaxsentan and valdecoxib) received a safety warning 20 days after their Notice of Compliance. Cerivastatin and lumiracoxib were withdrawn 23 and 48 days, respectively, after their respective safety warnings.

**Table 2:** Drugs withdrawn for safety reasons*

Generic name	ATC classification (third level)	Event; date (yr-mo-d)			Intervals, d			Reason for withdrawal
		NOC	First serious safety warning	Withdrawal	NOC to withdrawal	NOC to safety warning	Safety warning to withdrawal	
Pergolide	Dopaminergic	1991-06-27	None issued	2007-08-30	5908	NA	NA	Valvulopathy
Cisapride	Propulsive	1991-08-15	2000-02-14	2000-08-07	3280	3105	175	Cardiovascular events
Calcitonin (synthetic, salmon)	Antiparathyroid	1992-07-13	None issued	2013-10-01	7750	NA	NA	Malignancies
Remoxipride	Antipsychotic	1993-07-26	None issued	1994-03-14	231	NA	NA	Aplastic anemia
Nefazodone	Antidepressant	1994-04-27	2001-06-20	2003-11-27	3501	2611	890	Hepatotoxicity
Dexfenfluramine	Antiobesity	1996-07-09	1996-10-21	1997-09-15	433	104	329	Valvulopathy
Troglitazone	Glucose lowering	1997-05-09	None issued	2000-03-15	1041	NA	NA	Hepatotoxicity
Tolcapone	Dopaminergic	1997-10-08	None issued	1998-11-20	408	NA	NA	Hepatotoxicity
Cerivastatin	Lipid-modifying agent	1998-02-18	2001-07-16	2001-08-08	1267	1244	23	Rhabdomyolysis
Grepafloxacin	Quinolone antibacterial	1998-04-09	None issued	1999-10-26	565	NA	NA	Cardiovascular events
Trovafloxacin	Quinolone antibacterial	1998-12-04	None issued	2001-11-22	1084	NA	NA	Hepatotoxicity
Rofecoxib	Anti-inflammatory	1999-10-25	2002-04-19	2004-09-30	1802	907	895	Cardiovascular events
Sibutramine	Antiobesity	2000-12-28	2002-03-27	2010-10-08	3571	454	3117	Cardiovascular events
Gatifloxacin	Quinolone antibacterial	2001-01-09	2005-12-19	2006-06-29	1997	1805	192	Glucose metabolism disorders
Tegaserod	For constipation	2002-03-12	None issued	2007-03-30	1844	NA	NA	Cardiovascular events
Valdecoxib	Anti-inflammatory	2002-12-11	2002-12-31	2005-04-07	848	20	828	Skin reactions
Drotrecogin alfa	Antithrombotic	2003-02-20	None issued	2011-10-25	3169	NA	NA	Failure to show benefit
Efalizumab	Immunosuppressant	2005-10-24	2008-12-22	2009-02-22	1217	1155	62	Progressive multifocal leuko-encephalopathy
Lumiracoxib	Anti-inflammatory	2006-11-02	2007-08-16	2007-10-03	335	287	48	Hepatotoxicity
Sitaxsentan	Antihypertensive	2007-06-19	2007-07-09	2010-12-15	1275	20	1255	Hepatotoxicity
Ceftobiprole	β-Lactam antibacterial	2008-06-26	None issued	2010-04-16	659	NA	NA	Conduct of clinical trials
Idebenone	Psychostimulant	2008-07-23	None issued	2013-02-27	1680	NA	NA	Failure of efficacy studies
**Median (interquartile range)**					1271 (706–2876)	907 (196–1525)	329 (119–893)	

ATC = Anatomical Therapeutic Chemical classification system,10 NA = not applicable, NOC = Notice of Compliance.

* Ordered by date of approval.

At the third level of the Anatomical Therapeutic Chemical classification system, 2 classes—quinolone antibacterials and anti-inflammatories—were responsible for 3 drugs each; in addition, 2 of the drugs withdrawn were dopaminergic agents and 2 were for the treatment of obesity. Drugs were withdrawn primarily because of cardiovascular events, including valvulopathy (7 products), or because of hepatotoxicity (6 products) (Table 2).

## Interpretation

There was no difference in the percentage of drugs approved in the four 5-year periods that were eventually withdrawn from the market. The absence of any change indicates that the ability of the drug review system to detect serious safety issues and keep those drugs off the market did not change over the time period examined and similarly that there was no change in the rigour of the postmarket surveillance system. The discrepancy between the change in the number of reported adverse drug reactions (ADRs) and the change in the number of prescriptions filled suggests another explanation. From 1998 to 2011, the number of ADRs rose from 4663^11^ to 41 923,^12^ an increase of 799%, whereas the number of retail prescriptions rose only 107%, from 254 187 000^13^ to 524 952 000.^14^ If the postmarket surveillance system became better able to identify ADRs while the percentage of products withdrawn remained the same, then one possibility is that, over time, the premarket review system was becoming less stringent. Distinguishing between these competing explanations would require access to internal Health Canada documents to explore the reasons behind decisions about drug approvals and safety withdrawals.

The overall 4.2% figure for withdrawals in this study is generally in line with US and European values, although it is higher than the 2.9% (16 of 548 new molecular entities, the US equivalent of a new active substance) reported by Lasser et al.^15^ for the United States between 1975 to 1999 and lower than the 5.5% (16 out of 289) of new active substances approved by the European Medicines Agency between 1 January 1999 and 31 December 2009.^16^ The median time to withdrawal for the 16 drugs in the study by Lasser et al.^15^ was about 620 days, which is just under half the median of 1271 days in the study reported here. (Times in days from the study by Lasser et al.^15^ are approximations, since those authors reported times in years and fractions of years.) Five of the 16 drugs withdrawn in the United States, as reported by Lasser et al.,^15^ had a prior safety warning, with a median time between approval and the safety warning of 1241 days (interquartile range 1095–1460) and a median time between the safety warning and withdrawal of 1168 days (interquartile range 730–1497). The duration for the same intervals in Canada was 907 and 327 days, respectively. There are a number of possible explanations for these differences, including the mix of drugs approved in the different time periods, differences in the relative strengths and weakness of the US and Canadian drug regulatory systems, and differences in the willingness of the regulatory authorities to take action. Lasser et al.^15^ also reported that cardiovascular events (4 products) and hepatotoxicity (3 products) were the major reasons for withdrawals.

Just as important as the percentage of withdrawals is whether there has been a change in how many people are exposed to drugs before they are withdrawn. As an example, in 2003, the year before rofecoxib was removed from the market, it was the 10th most frequently prescribed medication in Canada.^17^ Unfortunately, there are no publicly available data to answer this question.

The 1271-day median time between Notice of Compliance and withdrawal emphasizes the need to be particularly cautious in prescribing new drugs early in their life cycle. Conversely, a prolonged period on the market is no guarantee of safety. It took over 21 years (7750 days) to recognize problems with synthetic salmon calcitonin. The short intervals between marketing approval and a serious safety warning and between a safety warning and withdrawal raise questions about both the pre- and the post-market safety evaluations: Was Health Canada unaware of the safety problems with sitaxsentan and valdecoxib when it approved these drugs? What changed in the few weeks between when a safety warning was issued about cerivastatin and lumiracoxib and when they were withdrawn? Eleven of the 22 drugs that were withdrawn had no prior safety warning. Was Health Canada unaware of any safety issues associated with these products before they were withdrawn? Health Canada documents describing what triggers a safety action are vague and give little indication about how decisions are made. For example, Health Canada states "The determination of the seriousness of risk (probability of health hazard and probability of occurrence) and urgency of risk communication is based on sound scientific judgement."^18^

This study had several limitations. The withdrawal of one drug (ceftobiprole) may have been for reasons other than safety. The definition of a serious safety warning was based on the way that Health Canada displayed the information (bold black print and/or boxed text), but the criteria that Health Canada used in deciding on its safety warnings and the emphasis that it placed on any particular safety issue are unknown. There were inconsistencies in the Health Canada databases. Some drugs identified as "new active substances" in the annual reports of the Therapeutic Products Directorate were not called "new active substances" in the Notice of Compliance online query website. The date on which a new active substance receives a Notice of Compliance is not necessarily the date on which the company decides to market the drug; therefore, the length of time the drug was available before it received a safety warning and/or was withdrawn may have been shorter than what is reported here. However, no information about when a drug is first sold is publicly available. Finally, follow-up for individual drugs ranged from slightly more than 3 years to over 23 years. Further safety withdrawals may occur, especially with more recently approved products. The effect on the results presented here would depend on the number of future withdrawals and when the particular products were approved.

This research emphasizes that a small but not insignificant number of drugs that are approved will eventually be found to be too unsafe to remain on the market. It also raises questions about the thoroughness of Health Canada's pre- and post-market evaluations of drug safety.
